# Characteristics of glycopeptide-resistant *Staphylococcus aureus* strains isolated from inpatients of three teaching hospitals in Warsaw, Poland

**DOI:** 10.1186/s13756-018-0397-y

**Published:** 2018-08-29

**Authors:** Ksenia Szymanek-Majchrzak, Andrzej Mlynarczyk, Grazyna Mlynarczyk

**Affiliations:** 1 Department of Medical Microbiology, Medical University of Warsaw, T. Chalubinskiego 5 Str, 02-004 Warsaw, Poland; 2 Department of Medical Microbiology, The Infant Jesus Teaching Hospital, Lindleya 4 Str, 02-005 Warsaw, Poland

**Keywords:** MLST, SCC*mec*, *Staphylococcus aureus*, Teicoplanin, TRSA, Vancomycin, VRSA

## Abstract

**Background:**

Vancomycin is still one of the most commonly used drug for treatment of severe methicillin-resistant *Staphylococcus aureus* (MRSA) infections. Vancomycin-resistant *S. aureus* (VRSA) strains are a serious danger for public health. This study aimed to characterize healthcare-associated MRSA (HA-MRSA) strains, resistant to at least one of glycopeptide antibiotics: vancomycin (VRSA) and/or teicoplanin (TRSA), isolated at three Warsaw hospitals over a period of 17-years (1991–2007).

**Methods:**

Among 600 HA-MRSA strains, isolated from patients with symptomatic infections, 47 were subjected to detailed analysis. In the study, mechanisms behind VRSA phenotypes were determined (E-tests, GRD-test, agar-dilution method and *van*A/B detection). Characteristics of selected isolates on molecular level: i) by detection of resistance genes *erm*A/*erm*B/*erm*C, *msr*A/*msr*B, *lin*A/*lin*A’, *aac*A-*aph*D, *aad*D, *aph*(3”)-IIIa; ii) SCC*mec*-typing and iii) MLST-typing was done.

**Results:**

In general population of studied strains, 11/47 (23.4%) were VRSA and 36/47 (76.6%) were resistant only to teicoplanin. All isolates exhibited *van*-independent mechanisms of resistance. Over 80% of isolates belonged to clonal complex CC8, with the following predominant sequence types (STs)/clones: ST247-IA/Iberian, ST241-III/Finland-UK, and ST239-III/Brazilian. Most of the isolated strains harboured *erm*A and *aac*A-*aph*D genes, encoding additional resistance to macrolides, lincosamides, streptogramin B, and majority of aminoglycosides. They occurred also in Polish VRSA/TRSA population over the period, which was subjected for analysis: an increase in MIC values for glycopeptides, evolution in terms of the level and extent of resistance, and genetic re-assortment in epidemic clones.

**Conclusions:**

VRSA strains isolated from patients hospitalized at three Warsaw teaching hospitals in Poland, over a period of 17-years do not pose a threat as potential donors of *van* genes in horizontal-gene transfer processes, but are constantly evolving and represent international epidemic clones.

## Background

Vancomycin is used in medicine for nearly six decades and despite that fact, it continues to be one of the key therapeutic options in the treatment of severe, live-threatening methicillin-resistant *Staphylococcus aureus* (MRSA) infections. The first emergence of cases of therapeutic failure concerning vancomycin, raised considerable concern among both microbiologists and clinicians. These cases were attributed mostly to an overuse of glycopeptide antibiotics, e.g. the years 1980–1996 saw a nearly 14-fold increase in the use of vancomycin in hospital settings in Western Europe and the US [1].

The 2018 European Committee on Antimicrobial Susceptibility Testing (EUCAST) criteria, currently used in Poland and the rest of Europe, define glycopeptide-resistant (VRSA, TRSA) *S. aureus* isolates as those with MIC values for vancomycin (VAN) and/or teicoplanin (TEI) of > 2.0 mg/L. Isolates, which have resistance mechanisms that are either associated with or independent of the VanA operon [2, 3] are considered to fulfil this criterion. The latter type of resistance mechanism has been decidedly more common ever since the VanA-negative strain variants were first described in the 1990s. As a result of the different resistance criteria used at the time, those VanA-negative strains were initially called vancomycin intermediate *S. aureus* (VISA) and heterogeneous VISA (hVISA) [4–6]. Their resistance phenotype is relatively low (vancomycin MIC of 4–8 mg/L), which is similar and determined by multiple genes (with strain Mu50 exhibiting altered expression of over 200 genes in comparison with vancomycin-sensitive strain N315) and involves mainly structural changes to bacterial cell wall; however, mutations have been also detected in global regulatory system genes, as well as genes associated with RNA synthesis, cell transport, and carbohydrate metabolism [1, 7–9]. In contrast, the first of two mentioned mechanisms of resistance, is characteristic for VanA-positive VRSA strains and is similar to the one reported in vancomycin resistant *Enterococcus* (VRE) [2, 3]. The first VRSA strain with VanA operon, was isolated in Michigan, US, in 2002. So far, there have been fewer than 20 clinical isolates of VanA-positive *S. aureus* reported in the literature, including at least 14 from the US [3, 10, 11]. In Europe there has been a single case of an infection with a highly resistant VRSA strain, which was reported in Portugal in 2013 [12]. Little is known about the prevalence and characteristics of VRSA in Poland, with few reports available on the subject [13].

Vancomycin-resistant *S. aureus* strains undeniably pose an important therapeutic problem. This was emphasized by the fact that during the February 25–27, 2017, Geneva World Health Organization meeting, world-class experts decided to include VRSA strains among the top 12 most dangerous pathogens of the, so-called, global priority pathogens list (global PPL) of antibiotic-resistant bacteria that pose a threat to public health [14]. These facts prompted us to evaluate vancomycin- and/or teicoplanin-resistant *S. aureus* strains isolated from three Warsaw teaching hospitals over a number of years, which would include determining the mechanism behind their VRSA phenotype and analysing selected isolates on the molecular level.

## Methods

### Bacterial strains

The studied material consisted of 47 (selected from 600 strains initially) clinical isolates of healthcare-associated methicillin-resistant *S. aureus* (HA-MRSA) exhibiting resistance to at least one of the two evaluated glycopeptide antibiotics: vancomycin and/or teicoplanin. The MRSA strains between No. 1 to 47 were isolated from patients hospitalized at one of three large Warsaw teaching hospitals, including one pediatric hospital, over a period of 17 years (1991–2007). The study population was obtained from the following hospital wards (No. of isolates provided in parentheses): surgery (10), hematology (9), orthopedics (8), ICU (5), oncology (4), dermatology (3), neonatology (3), gynecology (2), internal medicine (2), and transplantation ward (1). Strains were sampled mainly from wounds, skin, subcutaneous tissue infection swabs (29) and blood (12). Fewer isolates were sampled from eyes (3), bile (1), tracheal aspirate (1), and tracheostomy tube (1). In the study, the following reference strains were used: ATCC 29213 – methicillin susceptible *S. aureus* (MSSA), vancomycin susceptible *S. aureus* (VSSA); MR3 – MRSA, *mec*A^+^, VSSA; ATCC 700698 (Mu3) – MRSA, heterogeneous-VISA; ATCC 700699 (Mu50) – MRSA, VISA; WH 1268VA – VRE, *van*A^+^; WH 1266VB – VRE, *van*B^+^. The isolates were obtained from collection of the Department of Medical Microbiology, Medical University of Warsaw.

### Strain identification, screening tests and MIC values determination for vancomycin and teicoplanin

Automated strain identification with Gram-positive (GP) Vitek 2 identification card (bioMerieux, France) according to the manufacturer’s instructions was performed. Susceptibility to methicillin was determined with cefoxitin discs (FOX 30 μg, Oxoid), according to the current EUCAST recommendations; *mec*A gene was detected with the PCR technique [15].

The first-line screening test to determine sensitivity to vancomycin and teicoplanin with AST-P580 cassettes in Vitek 2 system (bioMerieux, France) was performed, MIC values for VAN and TEI were determined with standard E-tests (bioMerieux, France). All strains which occurred less susceptible (even in the range of sensitivity, MIC VAN/TEI values at least 1 mg/L or higher), were classified to the further investigations. In the next step E-test GRD (glycopeptide resistance detection), (bioMerieux, France) was performed according to manufacturer’s guidelines, as well as agar dilution method, according to EUCAST ISO 20776–1. Forty seven MRSA strains that were both: positive in the GRD gradient test (MIC value equal to 8 mg/L or higher for VAN or TEI), and with MIC value for VAN ≥3 mg/L (in diagnostic manner at least 4 mg/L) in agar dilution method, were classified as an appropriate materials for analysis in this study.

### Drug-resistance genes detection

Genes responsible for antibiotic resistance were detected through PCR technique with the appropriate primer pairs: *van*A, *van*B - VanA-1 and VanA-2, VanB-1 and VanB-2; *erm*A, *erm*B, *erm*C, *msr*A, *msr*B, *lin*A, *lin*A’ – ErmA-1 and ErmA-2, ErmB-1 and ErmB-2, ErmC-1 and ErmC-2, MsrAB-1 and MsrAB-2, LinAA’-1 and LinAA’-2, respectively; *aac*A-*aph*D, *aad*D, *aph*(3″)-IIIa – AacA/AphD-1 and AacA/AphD-2, AadD-1 and AadD-2, Aph(3″)-IIIa-1 and Aph(3″)-IIIa-2 [16–19].

### SCCmec (staphylococcal chromosome cassettes mec) characteristics

SCC*mec* types/subtypes, types of *ccr* gene complexes, and classes of *mec* gene complexes were determined according to the procedure described by Oliveira and Okuma [15, 20].

### Multilocus sequence typing

Multilocus sequence typing (MLST) was conducted on the base of seven housekeeping gene sequences evaluation (*arc*C, *aro*E, *glp*F, *gmk*, *pta*, *tpi*, *yqi*L), according to the procedure described by Enright [21]. The sequence types (STs) and clonal complexes (CCs) were determined by database available at http://saureus.mlst.net/.

Evaluated isolates were classified as individual MRSA clones based on the results of SCC*mec*, ST, and CC typing.

## Results

All 47 evaluated clinical isolates of HA-MRSA were both *van*A and *van*B negative and were resistant to single or both tested glycopeptide antibiotics; the MIC values manifested vancomycin resistance in case of 11 (23.4%) strains (VAN MIC = 4–8 mg/L, VRSA phenotype) and teicoplanin resistance in case of 45 (95.7%) (TEI MIC = 4–16 mg/L, TRSA phenotype), with 9 (19.1%) exhibiting simultaneous resistance to both glycopeptide antibiotics (VRSA/TRSA phenotype). Table 1 presents detailed data on vancomycin and teicoplanin MIC values, data interpretation, and the resistance phenotypes for all evaluated isolates.

**Table 1:** Vancomycin and teicoplanin MIC (mg/L) values for 47 HA-MRSA strains

MIC (mg/L)	No of strains (%), *n* = 47		Interpretation* VAN/TEI
	VAN^1^	TEI^2^	
0,016 – 0,5	0 (0%)	0 (0%)	S/S^3^
1,0	6 (12,8%)	1 (2,1%)	S/S
2,0	30 (63,8%)	1 (2,1%)	S/S
4,0	11 (23,4%)	28 (59,6%)	R/R^4^
8,0	0 (0%)	10 (21,3%)	R/R
16,0	0 (0%)	7 (14,9%)	R/R
32–256	0 (0%)	0 (0%)	R/R

Legend: ^1^*VAN* Vancomycin; ^2^*TEI* Teicoplanin; ^3^*S* Susceptible; ^4^*R* Resistant; *According to EUCAST, 2018

Vancomycin MIC values for all VRSA strains were equal to 4 or 8 mg/L. Table 2 contains characteristic of 11 studied VRSA strains (VRSA/TSSA – 2 strains and VRSA/TRSA – 9 strains) and presents vancomycin and teicoplanin MIC values for them. Table 2 also contains information on the year of strain isolation as well as on MLST STs, MLST CCs, and SCC*mec* types of the 11 VRSA strains. Additionally a potential MRSA affiliation into epidemic clones was also suggested. Moreover, the prevalence of the following drug-resistance genes was determined: *erm*A, *erm*B, *erm*C, *msr*A, *msr*B, *lin*A, *lin*A’ (encoding resistance to macrolides, lincosamides, streptogramin B), *aac*A-*aph*D, *aad*D, *aph*(3″)-IIIa (encoding resistance to various combinations of aminoglycoside antibiotics).

**Table 2:** Phenotypic and genotypic characteristics of 11 clinical VRSA isolates (VRSA/TSSA – 2 strains and VRSA/TRSA – 9 strains)

Nr of strain	Year of isolation	Source^1^	MIC_VAN/ TEI_^2^ (mg/L)	SCC *mec* type^3^	MLST^4^		Other resistance genes^7^	Presumable clone
					ST^5^	CC^6^		
MRSA-27	2003	H-2	4/4	IA	ST247	CC8	1, 2, 3, 4	Iberian
MRSA-31	2005	H-2	4/8	IA	ST247	CC8	1, 2, 3, 4	Iberian
MRSA-34	2005	H-3	4/16	IA	ST247	CC8	1, 3, 4	Iberian
MRSA-35	2005	H-3	4/16	IA	ST247	CC8	1, 3, 4	Iberian
MRSA-37	2005	H-3	4/16	IA	ST247	CC8	1, 3, 4	Iberian
MRSA-23	2003	H-2	4/16	III	ST241	CC8	1, 2, 3, 4, 5	Finland-UK
MRSA-43	2005	H-1	8/8	III	ST241	CC8	1, 3, 5	Finland-UK
MRSA-44	2005	H-1	4/8	III	ST241	CC8	1, 3, 5	Finland-UK
MRSA-45	2005	H-1	4/4	III	ST241	CC8	1, 3, 5	Finland-UK
MRSA-40	2005	H-1	4/2	IV	ST1	CC1	1	UD^8^
MRSA-22	1996	H-1	8/1	IV	ST30	CC30	1, 3, 4, 5	UD^8^

Legend: ^1^Source of strains: H-1, H-2, H-3, hospital number 1, 2, 3; ^2^*VAN/TEI* Vancomycin/teicoplanin; ^3^*SCCmec type* Staphylococcal chromosome cassettes *mec* type; ^4^*MLST* Multilocus sequence typing; ^5^*ST* Sequence type; ^6^*CC* Clonal complex; ^7^Other resistance genes: 1, *erm*A; 2, *erm*C; 3, *aac*A*-aph*D; 4, *aad*D; 5, *aph*(3”)-IIIa; ^8^ UD, undefined

Nine out of 11 vancomycin-resistant strains exhibited co-resistance to teicoplanin. These isolates resistant to both antibiotics belonged to ST247 and ST241 and were mostly isolated in 2005. ST247 strains were isolated at hospitals 2 and 3, whereas ST241 strains were isolated mainly at hospital 1. Two TSSA (teicoplanin susceptible *S. aureus*) isolates represented other STs (Table 2).

Table 3 presents the characteristics of 36 VSSA/TRSA isolates (VAN MIC = 1–2 mg/L; TEI MIC = 4–16 mg/L). Also in this case, the predominant strains belonged to ST247 and ST241. In total, the 36 VSSA/TRSA isolates represented 10 different STs.

**Table 3:** Phenotypic and genotypic characteristics of 36 *S. aureus* isolates susceptible to vancomycin and resistant to teicoplanin (VSSA/TRSA)*

ST^1^	CC^2^	SCC *mec* type^3^	MIC_VAN_^4^ (mg/L)	MIC_TEI_^5^ (mg/L)	Presumable CLONE	No. of strains	Other resistance genes found (No. of strains)	Year of isolation (No. of strains)
ST247	CC8	IA	1–2	4–8	IBERIAN	10	*erm*A (10), *erm*C (2), *aac*A*-aph*D (10), *aad*D (10)	1994(8), 2003(1), 2005(1)
ST241	CC8	III	1–2	4–16	FINLAND-UK	7	*erm*A (7), *lin*A(1), *aac*A*-aph*D (7), *aad*D (4), *aph*(3″)-IIIa (6)	2003(2), 2004(2), 2005(1), 2007(2)
ST239	CC8	III	2	4–8	BRASILIAN	5	*erm*A (5), *erm*C (2), *aac*A*-aph*D (5), *aph*(3″)-IIIa (4)	1991(1), 1994(4)
ST8	CC8	IV	1–2	4	UK EMRSA-2	4	*erm*A (1), *erm*C (1), *aac*A*-aph*D (1), *aad*D (2)	2004(1), 2005(3)
ST254	CC8	IV	2	4	HANNOVER	3	*erm*A (3), *aph*(3″)-IIIa (3)	1994(2), 2005(1)
ST461	CC5	I	2	4	UD^6^	2	ND^7^	1991(2)
ST2326	CC5	NT	2	4	UD^6^	2	*erm*C (2), *aad*D (2)	1991(2)
ST5	CC5	I	2	8	UK EMRSA-3	1	ND^7^	1991(1)
ST1	CC1	IV	2	4	UD^6^	1	ND^7^	2005(1)
ST2323	single	IA	2	8	UD^6^	1	*erm*A (1), *aac*A*-aph*D (1), *aad*D (1)	1994(1)

Legend: *According to EUCAST, 2018; ^1^*ST* Sequence type; ^2^*CC* Clonal complex; ^3^*SCCmec type* Staphylococcal chromosome cassettes *mec* type; ^4^*VAN* Vancomycin; ^5^*TEI* Teicoplanin; ^6^*UD* Undefined; ^7^*ND* Not detected

All evaluated strains (with two exceptions) exhibited one of the following SCC*mec* types: I/IA, III, and IV. Each strain belonged to one of the following types of the *ccr* gene complex: *ccr*1, *ccr*2, and *ccr*3 and to one of the following classes of the *mec* gene complex: A and B. These genotypes were detected in the following combinations: SCC*mec*-I/IA (1B), 19 isolates; SCC*mec*-III (3A), 16 isolates; and SCC*mec*-IV (2B), 10 isolates. Based on SCC*mec* types and STs of some isolates, their affiliation to international epidemic clones was proposed (Tables 2 and 3). The *erm*A, *erm*C, *lin*A/*lin*A’, *aac*A-*aph*D, *aad*D, and *aph*(3″)-IIIa genes were detected in 38/47 (80.9%), 10/47 (21.3%), 1/47 (2.1%), 34/47 (72.3%), 26/47 (55.3%), and 18/47 (38.3%) strains, respectively. None of these isolates harboured following genes: *erm*B, *msr*A, *msr*B. In case of four isolates, no tested genes were detected.

## Discussion

Around the world *S. aureus* strains of the VRSA phenotype associated with the VanA operon are isolated very rarely. This is most likely due to the fact that genetic selection of such a strain variant requires consecutive occurrence of several genetic events. Moreover, in order for the genes encoding glycopeptide resistance to be expressed, the recipient’s strain must be a mutant defective in terms of a type I restriction-modification (R-M) system. Another factor limiting the spread of *van*-positive *S. aureus* strains are mutations in the *ddl* gene, which encodes a D-Ala-D-Ala ligase [3]. Much more prevalent and characterized global widespread are variants with *van*-independent mechanism, which in the past were called VISA/hVISA. In this study, 11*. aureus* strains resistant to vancomycin were detected, which exhibited the VAN MIC value of 4–8 mg/L. None of the evaluated strains harboured the *van*A or *van*B genes, derived from vancomycin resistant enterococci. That means polish VRSA strains are probably derivatives of VISA/hVISA variants.

The first report of clinical VRSA isolates in Poland was published in 2002 and related only to *van*-independent mechanism variants. These reported strains had been isolated in 2000 (from blood and sputum of patient hospitalized at Oncology Centre in Warsaw) and in 2001 (from an asymptomatically colonized neonate at Warsaw pediatric hospital). The vancomycin and teicoplanin MIC values of these three isolates were 4 mg/L and 8 mg/L, respectively and these strains were very closely related [13]. The VRSA isolates described in this paper were isolated mainly in 2003 and 2005 (a total of 10/11 isolates) and represent consecutive VRSA strains occurring in Poland after the year 2002. Four strains of the ST241-CC8-SCC*mec*-III (3A) type were classified to the Finland-UK epidemic clone. This clone belongs to the same clonal complex as pandemic strain ST239-CC8-SCC*mec*-III (3A), but the spread of the ST241-CC8-SCC*mec*-III (3A) limited mostly to the United Kingdom and Finland [22]. Five out of the remaining VRSA strains encoded SCC*mec* type IA (1B) and, based on their sequence type (ST247) and clonal complex (CC8), were classified as belonging to the Iberian clone. The Iberian clone was extensively reported in the US as well as in many European countries (including Poland), especially in the 1990s [22–25]. After the year 2000, there was a considerable decrease in the proportion of these *S. aureus* variants as a causative factor of infections in humans. In Italy, this decrease was nearly 4-fold in comparison to the figures from 1990 to 1999 [23]. There were also reports of a considerable increase in vancomycin and teicoplanin MIC values for ST247-IA over the same period, which may be explained by antibiotic-induced pressure towards accumulation of point mutations and the resulting selection of variants characterized by a lower sensitivity or even phenotypic resistance to glycopeptides.

Among 36 isolates exhibiting resistance only to teicoplanin, there were mainly variants belonged to CC8 (ST247-IA, ST241-III, ST239-III, ST8-IV, and ST254-IV). Most of them (21, 58.3%) were isolated in the 1990s, which supports the data provided by Campanile [23]. Moreover, it is noteworthy that strains of common Brazilian clone ST239-CC8-SCC*mec*-III (3A) constituted a considerable proportion of the isolated TRSA strains, were all isolated in the 1990. Conversely, in the population of VRSA/TRSA strains isolated mainly in 2003 and 2005, the Brazilian clone was dominated by the Iberian and Finland-UK clones. It may suggest that ST239-III strains exhibit poorer adaptive properties towards selective glycopeptide pressure than ST247-IA and ST241-III strains.

Among all analysed VRSA/TRSA strains, those classified as part of the Iberian, Finland-UK and Brazilian clone, exhibited the *erm*A and *aac*A-*aph*D genes. These genes are specific for nosocomial MRSA strains. Moreover, all Iberian clone isolates had also the *aad*D gene, which is mostly found in the pUB110 plasmid, characteristic for SCC*mec* types IA and II [26].

## Summary and conclusions

In summary, all isolates among analysed VRSA and/or TRSA strains exhibited antimicrobial resistance in the mechanism independent of Van operon. Over 80% of isolates belonged to CC8, constitute a group belonged mainly to the following sequence types/clones: ST247-IA/Iberian, ST241-III/Finland-UK, and ST239-III/Brazilian, which harbours *erm*A and *aac*A-*aph*D genes. These findings demonstrate that strain variants predominant in 1990s, were resistant only to teicoplanin and were represented mainly by the Iberian and Brazilian clones, whereas 91% of the VRSA/TRSA strain variants were isolated in the twenty-first century and belonged predominantly to the Iberian or Finland-UK clones. ST247-IA strains, which are chronically present in Polish VRSA/TRSA population, can be suspected to generate easily glycopeptide-resistant variants under selective pressure of vancomycin. Conversely, the Brazilian clone (ST239-III), commonly found in Europe and the rest of the world but less adaptable to antibiotic pressure, has been replaced by representatives of the Finland-UK clone (ST241-III), which was only isolated locally before. In conclusion, it can be stated, that VRSA strains isolated from patients hospitalized at three large Warsaw teaching hospitals in Poland, over a period of 17 years (1991–2007) do not pose a threat as potential donors of *van* genes in horizontal gene transfer processes, but are constantly evolving and represent international epidemic clones.

## Abbreviations

CC: Clonal complex
GRD: Glycopeptide resistance detection
HA-MRSA: Healthcare-associated MRSA
MLST: Multilocus sequence typing
MRSA: Methicillin resistant *Staphylococcus aureus*
MSSA: Methicillin-susceptible *S. aureus*
PPL: Priority pathogens list
SCC*mec*: Staphylococcal chromosome cassettes *mec*
ST: Sequence type
TEI: Teicoplanin
TRSA: Teicoplanin resistant *S. aureus*
TSSA: Teicoplanin susceptible *S. aureus*
VAN: Vancomycin
VISA/ h-VISA: Vancomycin intermediated *S. aureus*/ heterogeneous-VISA
VRE: Vancomycin resistant *Enterococcus*
VRSA: Vancomycin resistant *S. aureus*
VSSA: Vancomycin susceptible *S. aureus*
